# 180°-Talushalsluxationsfraktur, Hawkins-Typ III

**DOI:** 10.1007/s00113-020-00898-0

**Published:** 2020-10-12

**Authors:** D. Völk, P. Biberthaler, H. Wegmann

**Affiliations:** Klinik und Poliklinik für Unfallchirurgie, Klinikum rechts der Isar, Technische Universität München, Ismaninger Straße 22, 81675 München, Deutschland

## Anamnese

Ein 26-jähriger Patient stürzte im Rahmen seiner beruflichen Tätigkeit auf einer Baustelle aus ca. 6 m in einen Kellerschacht. Dabei landete er im Sinne eines Hyperextensionstraumas auf einer Treppenstufe. Der Patient beklagte starke Schmerzen im Bereich des rechten Sprunggelenks, sodass eine Ruhigstellung im SAM-Splint erfolgte.

## Befund

**Figure 1:**
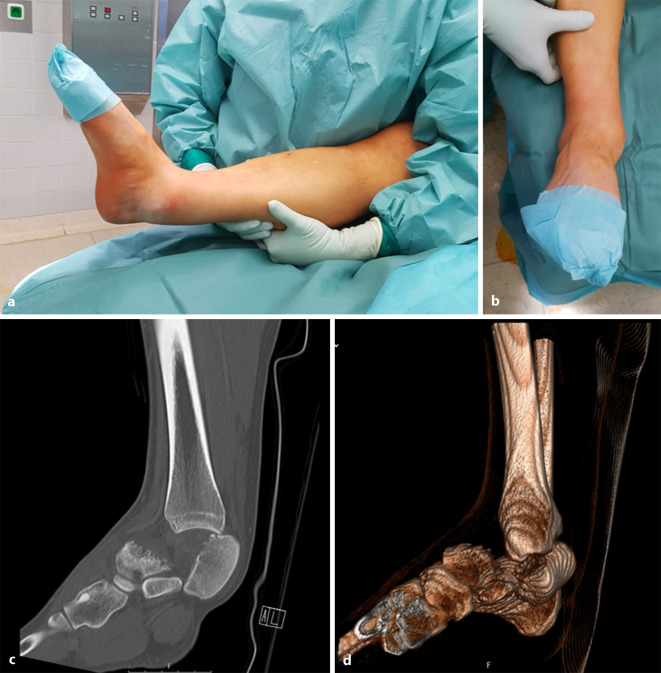

In der klinischen Untersuchung fiel am rechten Sprunggelenk eine deutlich sichtbare Fehlstellung mit Fragmentdruck im Bereich des Malleolus medialis auf (Abb. 1a,b). Die A. dorsalis pedis und A. tibialis posterior wiesen in der Untersuchung mittels Gefäßdoppler ein biphasisches Signal auf. Anamnestisch bestanden weder Vorerkrankungen noch ein Nikotinabusus.

## Bildgebung

Aufgrund des Unfallmechanismus mit Sturz aus über 3 m Höhe wurde eine Polytraumaspirale durchgeführt. Hier zeigte sich am rechten Sprunggelenk eine Talusfraktur mit Luxation des Taluskörpers nach posteromedial hinter den Malleolus medialis (Abb. 1c,d). Darüber hinaus ergab sich kein Hinweis auf eine weitere Traumafolge.

## Diagnose

Talusluxationsfraktur, Hawkins-Typ III, rechtsseitig.

## Therapie

Nach der Diagnosestellung wurde umgehend die Indikation zur Reposition in Intubationsnarkose unter Relaxierung, mit Konversion auf eine offene Reposition inklusive, gestellt.

**Figure 2:**
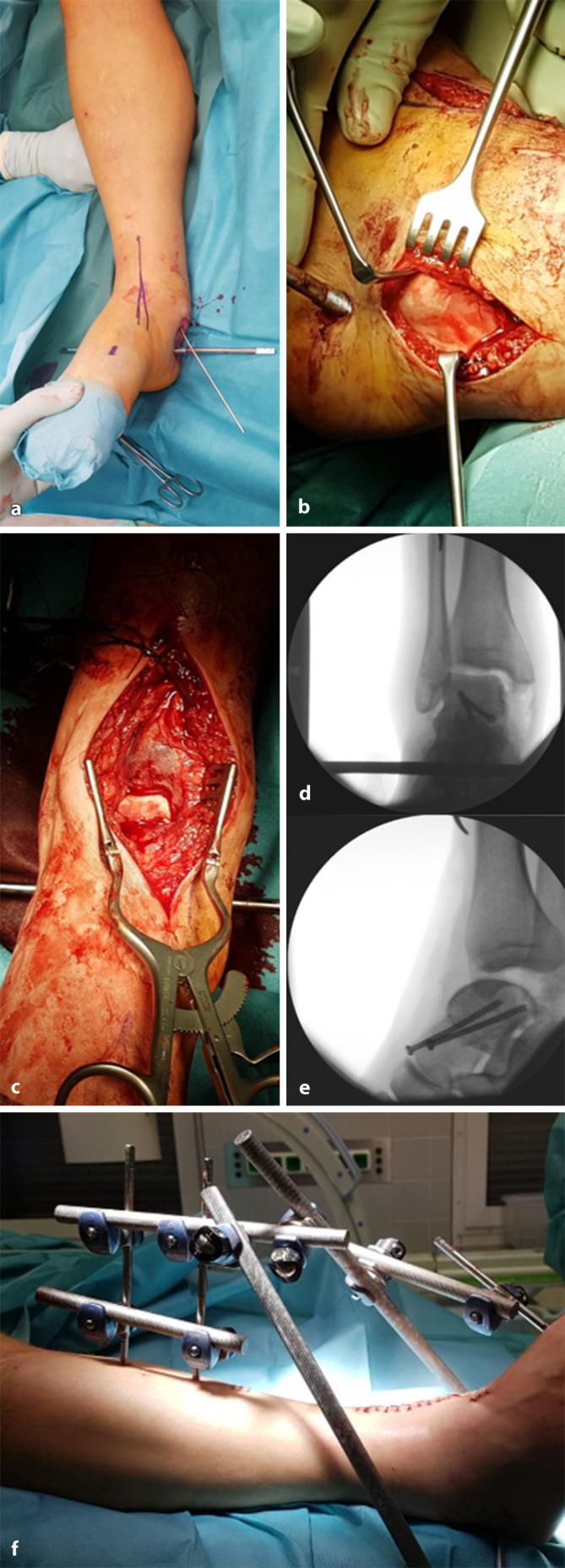

Zur Verbesserung der Zugkraft wurden in die Tibia ein Pin sowie in den Kalkaneus ein Steinmann-Pin eingebracht, aber auch hierunter war keine geschlossene Reposition möglich. Da sich die Großzehe in Flexion fixiert zeigte, bestand der Verdacht, dass eine Sehne als Weichteilinterponat vorhanden sein könnte. Daher wurde auf ein offenes Verfahren gewechselt. Zunächst wurde ein anteromedialer Zugang (Abb. 2a) geschaffen. Dabei zeigte sich der Corpus tali, komplett um 180 Grad verdreht, dorsal des Malleolus medialis liegend. Weitere Repositionsversuche auch unter Zuhilfenahme eines 2,5-mm-Kirschner-Drahtes im Corpus tali blieben erfolglos. Unter Bewahrung einer ausreichenden Hautbrücke wurde ein zusätzlicher posteromedialer Zugang geschaffen. Die für die Talusversorgung wichtigen Gefäßäste aus der A. tibialis posterior wurden geschont (Abb. 2b). Die Sehne des M. flexor hallucis longus interponierte als Repositionshindernis wurde befreit. Anschließend gelang die Reposition mittels Zug am Steinmann-Pin, Druck mit einem Stößel auf den Talus und maximaler Kniebeugung sowie Plantarflexion des Fußes zur Entspannung der Achillessehne (Abb. 2c). Die Fraktur wurde mittels zweier kanülierter 4,0-mm-Kurzgewindeschrauben von anteromedial und über eine Stichinzision von anterolateral versorgt (Abb. 2d). In der anschließenden Durchleuchtung zeigte sich eine Subluxationsstellung im OSG nach ventral (Abb. 2e). Daher wurde zusätzlich ein gelenkübergreifender Fixateur externe angebracht (Abb. 2f).

## Verlauf

Der postoperative Verlauf gestaltete sich im Weiteren unter Entlastung problemlos; die periphere Durchblutung, Sensorik und Motorik zeigten sich stets intakt. Eine CT-Untersuchung am 2. postoperativen Tag zeigte ein gutes Repositionsergebnis und eine regelrechte Schraubenlage. Der Fixateur externe wurde für insgesamt 7 Wochen belassen. Intraoperativ zeigte sich das OSG bandstabil. Der Patient wurde unter zunehmender Aufbelastung mobilisiert; die Frakturheilung erfolgte ohne radiologische Zeichen einer frühen Osteonekrose (Abb. 2f). In der Kontrolle 15 Wochen nach Op. zeigten sich eine ROM von 20° in Plantarflexion und Dorsalextension sowie klinisch kein Anhalt für eine ligamentäre Instabilität des Sprunggelenks.

## Fallanalyse

Die Talusfrakturen stellen mit ca. 2,5 % aller Frakturen [1, 2] eine seltene Entität dar, wobei Hawkins-Typ-III-Frakturen ca. 32 % aller Talusfrakturen darstellen [3]. Die CT-Untersuchung ist die Bildgebung der Wahl, insbesondere bei komplexen Frakturen und zur Detektion von Begleitverletzungen [4, 5]. Nach der Diagnose der Luxationsstellung wurde der sofortige Versuch einer geschlossenen Reposition der Talusluxationsfraktur durchgeführt. Diese war jedoch – wie meistens bei Typ-III- und Typ-IV-Frakturen – nicht erfolgreich [3]. Die geschlossene Reposition bei Talusluxationsfrakturen gelingt nur seltenen. Um Weichteil- und neurovaskuläre Schäden zu vermeiden, ist aber eine rasche Reposition unbedingt anzustreben [6, 7].

Die Entscheidung zur Konversion auf ein offenes Vorgehen sollte bei frustranem geschlossenem Repositionsversuch zeitnah getroffen werden, um eine weitere Kompromittierung der Weichteile zu verhindern [3, 6, 8]. Trotz guter operativer Exposition über einen anteromedialen Zugang und des Einsatzes von Repositionshilfen (K-Draht, Steinmann-Pin) konnte in diesem Fall keine Reposition der Fraktur erreicht werden. Die interponierte Sehne des M. flexor hallucis longus konnte nur über einen zusätzlichen dorsomedialen Zugang adressiert werden. Zusätzlich erwies sich die Lücke zwischen Kalkaneus und Tibiahinterkante als zu klein, um den um 180° verdrehten Talus trotz adäquater Distraktion zwischen Kalkaneus und Tibia und direktem Druck zu reponieren. Hinzu kam, dass der Talus aufgrund der straffen Verbindung mit dem Lig. deltoideum nur eine geringe Mobilität aufweist, was die Repositionsmanöver zusätzlich erschwert. Als entscheidendes Repositionsmanöver erwies sich die vollständige Beugung im Kniegelenk, die zu einer Entlastung der Achillessehne führte, wodurch sich die Distanz zwischen Tibiahinterkante und Kalkaneus vergrößerte und die Reposition möglich wurde. Es erfolgte eine direkte Ausversorgung der Fraktur mittels Schraubenosteosynthese [9]. Nach Rekonstruktion der Gelenkkapsel und Verschluss der Weichteile wurde eine dynamische Untersuchung des OSG unter Bildwandlerkontrolle durchgeführt. Hier fiel eine Instabilität nach ventral auf, weshalb ein gelenkübergreifender Fixateur externe in Neutralstellung des OSG angelegt wurde. Hierunter konnte eine Weichteilkonsolidierung mit stabilem Bandapparat in der Nachkontrolle erreicht werden [8].

## Fazit für die Praxis

- Bei Talusluxationsfrakturen ist eine möglichst sofortige Reposition entscheidend, daher zeitnahe Konversion geschlossener Reposition auf eine offene Reposition.
- Bei Vorliegen einer Hyperflexion von Zehen sollte, aufgrund eines mit hoher Wahrscheinlichkeit vorliegenden Weichteilinterponats, die Fraktur primär offen reponiert werden; dies ermöglicht eine schonende Reposition unter Vermeidung von iatrogenen Knorpelschäden.
- Konsequente Schonung des Lig. deltoideum bei posteromedialem Zugang und Reposition zur Aufrechterhaltung der Blutversorgung und Verminderung der Nekrosegefahr.
- Bilateraler Zugang bei komplexen Luxationsfrakturen zu Verbesserung der Visualisierung und Identifikation möglicher Repositionshindernisse.
- Maximale Flexion des Kniegelenks kann die Reposition bei dorsalen Luxationen durch Entspannung der Achillessehne erleichtern.
- Dynamische Untersuchung des Sprunggelenks nach Reposition und Osteosynthese zur Detektion möglicher Begleitverletzungen.
- Fixateur externe zur Ausversorgung einer Sprunggelenkinstabilität bei begleitender kritischer Weichteilsituation.

## Abkürzungen

A.: Arteria
CT: Computertomographie
GCS: Glasgow Coma Scale
HWS: Halswirbelsäule
Lig.: Ligamentum
M.: Musculus
OSG: Oberes Sprunggelenk
RoM: „Range of motion“

## References

[1] Lin S, Hak DJ (2011). Management of talar neck fractures. Orthopedics.

[2] Fournier A (2012). Total talar fracture—Long-term results of internal fixation of talar fractures. A multicentric study of 114 cases. Orthop Traumatol Surg Res.

[3] Vallier HA (2015). Fractures of the talus: state of the art. J Orthop Trauma.

[4] Dale JD, Ha AS, Chew FS (2013). Update on talar fracture patterns: a large level I trauma center study. AJR Am J Roentgenol.

[5] Williams T (2012). Total talar fracture—inter- and intra-observer reproducibility of two classification systems (Hawkins and AO) for central talar fractures. Orthop Traumatol Surg Res.

[6] Boack DH, Manegold S, Haas NP (2004). Therapiestrategie bei Talusfrakturen. Unfallchirurg.

[7] Whitaker C, Turvey B, Illical EM (2018). Current concepts in Talar neck fracture management. Curr Rev Musculoskelet Med.

[8] Maher MH (2017). The acute management and associated complications of major injuries of the talus. JBJS Rev.

[9] Thordarson DB (2011). Talusfrakturen. Unfallchirurg.

